# Maternal deaths due to eclampsia in teenagers: Lessons from assessment of maternal deaths in South Africa

**DOI:** 10.4102/phcfm.v12i1.2305

**Published:** 2020-07-09

**Authors:** Jagidesa Moodley, Nnabuike C. Ngene

**Affiliations:** 1 Women’s Health and HIV Research Unit, Department of Obstetrics and Gynecology, Faculty of Health Sciences, University of Kwa-Zulu Natal, Durban, South Africa; 2 Department of Obstetrics and Gynaecology, Klerksdorp Hospital, Klerksdorp, South Africa; 3 Department of Obstetrics and Gynaecology, School of Clinical Medicine, Faculty of Health Sciences, University of the Witwatersrand, Johannesburg, South Africa

**Keywords:** teenage pregnancies, eclampsia, clinical lessons, obstetrics, gynaecology

## Abstract

**Background:**

Eclampsia remains a major cause of maternal mortality, particularly in teenage pregnancies. Healthcare professionals providing antenatal must regard teenagers as a high risk group for the pre-eclampsia-eclampsia syndrome.

**Setting:**

Data extracted from the South African Saving Mothers Report: 2014–2016.

**Aim:**

To establish the clinical details in teenage maternal deaths owing to eclampsia.

**Method:**

Retrospective review of the case records and maternal death assessment forms of teenagers that died due to eclampsia during 2014–2016.

**Results:**

There were 47 teenagers (aged 14 to 19 years) who died from eclampsia. Of these 18 out of 47 (38%) deaths occurred in the post-partum period. Forty (85.1%) of the patients had antenatal care. Three (6.4%) had post-partum eclampsia, and of the remaining 44 of the 47 (93.6%), the gestational age at first occurrence of a seizure ranged from 25 to 39 weeks. The blood pressures at the time of seizure ranged from systolic of 131 to 210 mmHg and diastolic of 89 to 130 mmHg. The commonest final causes of death were intracerebral haemorrhage associated with severe hypertension and multi-organ failure. Avoidable factors included transport delays, referral to the wrong levels of health care and poor care by health professionals.

**Conclusion:**

Teenage pregnancy is a risk factor for eclampsia-related death; awareness of borderline elevations of blood pressure levels from baseline values (prehypertension levels) and taking following national guidelines on the management of hypertensive disorders of pregnancy will decrease deaths from eclampsia.

## Introduction

**FIGURE 1:**
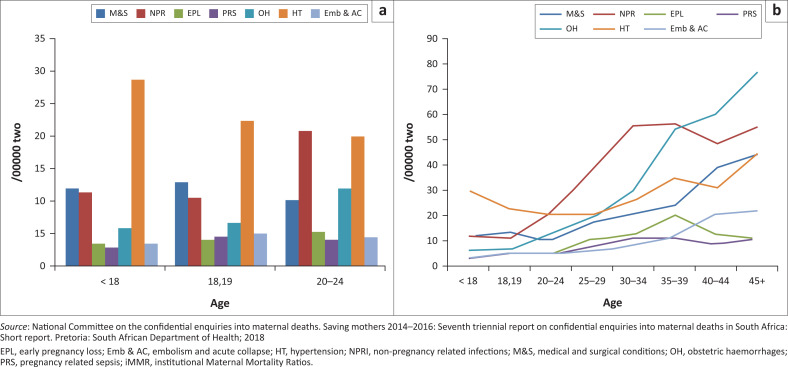
(a) Age (in years) per underlying cause of maternal death … the bar graph (b) institutional maternal mortality ratios per underlying cause of death.

Teenage pregnancies impact negatively on an individual’s education, employment, earning capacity and the well-being of their children.^1,2,3^ In addition, most teenage pregnancies are unintended, unwanted and are associated with high rates of morbidity and mortality, unsafe miscarriage and low birthweight babies.^1,2,3^ Recently, the Saving Mothers Report 2014-2016 of the National Committee on Confidential Enquiries into Maternal Deaths (NCCEMD) in South Africa (SA) found that when institutional Maternal Mortality Ratios (iMMR) related to maternal age are plotted against the underlying cause of death, there is a high rate of mortality in women aged ≤ 24 years.^1,2,3,4^ Hypertensive deaths owing to pregnancy accounted for large proportion of deaths in this age group. Furthermore, deaths from eclampsia (seizures associated with high blood pressure and proteinuria) occurred in teenagers (Figure 1).^4^

The 2017 annual report of the NCCEMD also indicates high rates of deaths from eclampsia in teenage pregnancies.^5^ Most teenagers initiate antenatal care in the second half of pregnancy and risk features of pre-eclampsia (PE) such as obesity and primiparity are not identified and preventative measures such as low-dose aspirin are not instituted. The aim of this study was to establish the clinical details in teenage maternal deaths owing to eclampsia.

## Methods

### Study design

Retrospective clinical chart reviews of teenage pregnancies that died due to eclampsia and pre-eclampsia during the period 2014 to 2016 in SA were conducted.

### Setting

Data were extracted from the NCCEMD database from 2014 to 2016. Deaths occurred at public and private facilities in South Africa.

### Data collection

The documents reviewed included patient hospital records, the maternal death notification forms and the assessment sheets of independent maternal death assessors. All documents were reviewed by the author, utilising methods previously described by NCCEMD.^4^ The following data were collected for each patient: maternal age, parity, duration of pregnancy at time of admission, blood pressure level at time of emergency admission, antenatal blood pressure levels if available, and evidence of the HELLP syndrome (haemolysis, elevated liver enzymes and low platelet counts). Data on foetal outcomes were also noted. Hypertension in pregnancy was defined as a blood pressure (BP) level of 140 mm Hg systolic and a diastolic of 90 mm Hg taken on two occasions, 4 h apart, in a previously normotensive patient; eclampsia was defined as convulsions or coma associated with hypertension and proteinuria in pregnancy.^6^ The management of eclampsia followed the recommendations of the South African Maternity Guidelines for Clinics and District Hospitals.^6^ Teenage pregnancy was defined as one occurring in the age group ≤ 19 years.

### Data analysis

Data capturing, checking and analysis were performed by the authors. Normally distributed continuous variables are reported as mean while categorical variables are reported as frequencies and percentages. Data are presented according to the format utilised and/or recommended by the National Committee on Confidential Enquiries into Maternal Deaths (NCCEMD) in South Africa.

### Ethical consideration

This article followed all ethical standards for a research without direct contact with human or animal subjects.

## Results

There were 47 cases of eclampsia in adolescents (aged ≤ 19 years) in the three-year study period and 10 were < 16 years (Table 1). Forty-two were tested for HIV and six (12.8%) were HIV positive; 40 patients had antenatal care and of these, 35 had booked for antenatal care after 20 weeks gestational age. Approximately 38% (*n* = 18) were undelivered and the caesarean delivery rate was 32% (*n* = 15).

**TABLE 1:** Teenage pregnancies: Clinical characteristics.

Variable		Number	%
**Age (years)**		**47**	**-**
14		3	6.4
15		2	4.2
16		5	10.6
17		4	8.5
18		20	42.6
19		13	27.7
Age range		14–19 years	-
**HIV status**		**47**	**-**
HIV infected		6	12.8
HIV uninfected		36	76.6
Unknown		5	10.6
**CD4 count**		**6**	**-**
> 300 cells/mL		3	50
Unknown		3	50
**Antenatal care (gestational age: weeks)**		**47**	**-**
< 20		5	10.6
> 20		35	74.5
Unbooked/no antenatal care		7	14.9
**Mode of delivery and fetal outcome**		**47**	**-**
Undelivered		18	38.3
Vaginal delivery		14	29.8
Alive		10	71.4
Stillbirth		4	28.6
Caesarean delivery *Includes 2 hysterotomies		15	31.9
Alive		7	46.7
Stillbirth		8	53.3
**Gestational age (weeks) at time of seizure**		**47**	**-**
Range		25–39	-
Mean		34 weeks	-
**BP (mmHg) at time of seizure**		**47**	-
Systolic			
Highest	210 mmHg		
Lowest	131 mmHg		
Mean	165 mmHg		
Diastolic			
Highest	130 mmHg		
Lowest	89 mmHg		
Mean	90 mmHg		
**Post-partum eclampsia**			
Aged 18 years	7 days following delivery: BP on discharge: 129/78 mmHg BP in labour: 138/62 mmHg.		
Aged 14 years	Known epileptic, developed elevated BPs >140/100 mm Hg and severe pre-eclampsia.		
Aged 19 years	Severe hypertension – discharged. On the 5th post-delivery day had convulsions and collapsed at home.		

BP, blood pressure.

The mean systolic and diastolic BP levels were 160 and 90 mm Hg respectively. However, there were eight patients who had systolic BPs < 140 mm Hg recorded at the time of emergency admission to hospital. There were 12 (25.5%) patients who had elevations and/or slight rises in BP from the basal BP levels (120 to 139/85 to 89 mm Hg or an increase in diastolic of 15 mm Hg and systolic of 30 mm Hg) during antenatal visits, on one or more occasions.

Three patients had post-partum seizures, and all had avoidable factors (Table 2). A patient with severe hypertension was discharged without adequate stabilisation of her elevated BP levels (> 160/110 mmHg) and had convulsions at home on the fifth day post-partum. The other two had slightly elevated BP at time of hospital discharge but antihypertensives were not provided at the time of discharge from hospital.

**TABLE 2:** Avoidable factors.

Avoidable factors	Number	%
**Patient orientated**		
Unbooked/No antenatal care	5	10.6
Declined advice	2	4.3
**Administrative issues**		
Ambulance delays	14	29.8
Referred to wrong level of health care facility	8	17.0
Shortage of ICU beds	4	8.5
Barriers to referrals – poor advice	4	8.5
**Health care professional issues**		
Failure to recognise ‘borderline’ slightly elevated BP levels at clinic level, intrapartum, and post-partum (both levels of diastolic 80 to 89 mm Hg and systolic 130 mm Hg to 140 mm Hg or rise in diastolic of 15 mm Hg or systolic of 30 mm Hg)	12	25.5
Management at inappropriate level of care – District hospital	6	12.8
Poor quality management/delay in management/early hospital discharge	4	8.5

ICU, intensive care unit; BP, blood pressure.

The exact final cause of deaths was known in 18 patients. In 29 cases the cause was unknown; the assessors thought that the cause of death in eight patients who died in intensive care units (ICUs) was due to intracranial pathology; four patients died in ambulances during transfer; one post-mortem showed an intracranial haemorrhage; the other 16 cases died of multi-organ failure, including respiratory distress and renal failure or a combination of these complications.

The main issues shown in Table 2 were a delay in ambulance services, referral to the wrong level of health care and the shortage of ICUs.

## Discussion

### Main findings

There were 47 deaths resulting from eclampsia in teenagers over a three-year period in South Africa, a low- to middle-income country (LMIC). Twelve of the 40 patients who had had antenatal care (Table 1) were found to have evidence of borderline elevations of blood pressure levels of between 120 and 139 mm Hg systolic and/or 80 to 89 mm Hg on one or more antenatal visits.

There were issues of considerable delays in arrival times of the ambulance at primary health care level for emergency referrals. Additionally, referrals to an inappropriate level of care occurred in a number of instances, such as patients being referred to a district hospital instead of a regional hospital, which resulted in four patients delivering in ambulances during transfer and a death in the casualty department at a district hospital in another instance.

The great majority of teenagers who died suffered avoidable factors mainly at administrative and healthcare provider levels in respect of clinical management at wrong level of care (as district hospitals in our setting should not be managing high-risk patients such as pre-eclampsia with severe features or eclampsia), failure to obtain advice and delay in providing emergency resuscitative management.

### Interpretation of findings

The findings of this relatively detailed clinical review of hospital records of teenagers who died from eclampsia are supported by the findings of the Saving Mothers Report 2014-2016 and the 2017 Saving Mothers Annual Report.^4,5^ These reports found that women in the age group below 20 years and above the age of 35 years contribute substantially to the numbers of deaths owing to hypertensive disorders of pregnancy (HDP) (Figure 1).

### Maternal age

The current study shows that most of the teenagers died from complications such as cerebral haemorrhage and/or cerebral oedema. That teenagers, and in particular teenage primigravidae, are more likely to develop HDP was reported from both high income countries and LMICs.^7,8,9,10,11^ The current study also highlights the negative impact on the large number of deaths from eclampsia in this age group in a LMIC where teenage pregnancies are still prevalent. Statistics South Africa reported on the number of birth registrations (*n* = 897 750), of which 98 445 (10%) were under the age of 19 years.^12^

Maternal age is one of the most important risk factors for eclampsia and women under the age of 20 years have a two- to six-fold higher risk in comparison with older women.^7,8,9,10,11^ The present study confirms that teenage pregnancies are at risk of eclampsia and maternal mortality. In a study in Tanzania (2012), 48.7% of women with a diagnosis of eclampsia were younger than 20 years and 43.7% had normal BP levels,^8^ while in a more recent study carried out in Colombia by Olay-Garay et al., 28.3% of cases of eclampsia were adolescents compared to 3% in adults, and although the BP levels were higher in the older group, they were not significantly different.^11^

### Blood pressure levels

The levels of BP play an important role in both the detection of hypertension and its management. There were 12 cases of rises in BP levels from the basal levels which were between 120 and 139 mm Hg systolic and/or diastolic 80 and 89 mm Hg in teenagers which, if acted upon, might have prevented eclampsia. Such actions include frequent monitoring of BP levels to detect further increases, checks for deranged laboratory tests for pre-eclampsia and monitoring for foetal compromise. Current guidelines for definitions of hypertension in pregnancy do not take into account maternal age and given our findings and those of Olaya-Garay et al., consideration should be given to the threshold rises in BP from basal levels in teenage nulligravidae, especially in LMICs, where adolescent pregnancies are prevalent.^11,12^ Studies done in South Africa show that a standard technique and BP devices validated in pregnancy should be used for BP measurements.^13^ Any elevation in BP or increases from basal BP levels should be repeated within 15 min and patients should be referred to the appropriate health facility timeously using the national maternity care guidelines.^6^

There is also evidence from the charts reviewed that eclampsia may occur abruptly. In the cases in which elevated borderline BP measurements were recorded, six patients developed eclampsia within five to seven days. Similarly, there were three cases of post-partum eclampsia in which BP levels were again slightly elevated during the intrapartum period or in the immediate post-partum period who returned within seven days of hospital discharge with convulsions having occurred at home. This suggests that eclampsia can and does develop rapidly; health care professionals must be made aware of this and ensure that BPs are measured appropriately and that antihypertensive medications are not only prescribed but that patients and their families, if possible, should be given full information about the warning signs of eclampsia and actions they should take if they experience symptoms and signs of a hypertensive crisis. In fact, the SA national guidelines for the management of HDP recommend that all patients with severe hypertension should be kept in hospital for at least 72 h for ‘BP stabilization’ following delivery and that antihypertensive agents should not be stopped abruptly but the dosages of the drug should be tailed off over a period of time. There are reports from Colombia that BP levels at the time of convulsions associated with eclampsia are lower than those of their adult counterparts.^8,9^ The present study reviewed detailed reports only of teenagers who died from eclampsia and therefore cannot comment on this, although there is evidence of BP values of greater than 140 mmHg systolic in eight patients. A study from Colombia reported that 26% had BP levels greater than 140/90 mm Hg and that 40% were younger than 20 years.^10^ This needs a more detailed study as BP measurements immediately after convulsions may be affected by a variety of medications which the patients received at a clinic/referring facility.

All teenagers in this study who had died, were primigavidae. It thus makes the case for raising awareness that both factors together (maternal age < 19 years in this study and first pregnancies) warrant consideration for the use of a low dose aspirin (LDA) for the prevention of PE. Although most booked for antenatal care after 20 weeks of gestation, some did start antenatal care before 20 weeks gestational age and in the latter group, LDA may still be useful. There is also evidence that calcium supplementation is effective in the prevention of HDP in countries with dietary calcium deficiencies.^14,15^

### Limitations and strengths

This was a retrospective chart review and dependent on documentation provided and the possible bias of the maternal death assessors and/or reviewers. There may have been poor documentation and failure to summarise patient case records, so the assessor may have misinterpreted the notes.

This study also defined teenage pregnancy as occurring in those ≤ 19 years and this definition may differ from that of other studies, which define adolescent pregnancies as being up to 22 years of age.^16^

Another limitation was the fact that this report confines itself to deaths from eclampsia. This was because the numbers who died from PE were in the single figures (*n* = 4) and did not have documentation of complete clinical information, and it was thought that no clear conclusions could be obtained from these cases. The occurrence of other co-morbidity such as abruption placenta and the severe pre-eclampsia may have also contributed to the mortality.

Despite the above limitations, the large number of deaths from eclampsia among teenagers in a three-year period is sufficient strength to make appropriate and practical recommendations in respect of teenage pregnancies.

## Recommendations

### General recommendations

- Reproductive Health Education involving male and female learners must be instituted at secondary level educational institutions.
- Community awareness of the social, economic, educational impact of teenage pregnancies must be strengthened.
- Contraceptive agents of all types must be freely available at secondary and tertiary educational facilities.
- Consideration must be given to easy access to termination of pregnancy services for those who request termination after counselling, especially for teenagers.

### Specific recommendations

1. Teenage pregnancies must be regarded as being at risk of PE and eclampsia. Therefore, pregnant teenagers should be seen at high-risk clinics and follow the basic antenatal care (BANC) Plus antenatal programme.
2. There is a need to improve the quality of antenatal care provided to patients with raised BP levels from baseline during the antenatal, intrapartum and post-partum periods. Severe hypertension must be managed as an emergency. This can be done by modifying clinical guidelines and/or protocols on management of HDP and by clinical specialists undertaking outreach visits to strengthen training at primary health care level.
3. Pregnant teenagers should be encouraged to attend antenatal clinics prior to 16 weeks gestation so that LDA and calcium supplementation can be initiated in the early second trimester.

### Future research

Strong consideration should be given to establish whether or not the definition of hypertension, particularly in teenagers, should include any rise in BP levels from 120 to 139 systolic and/or 80 to 89 mm Hg diastolic or to include any rise of 30 mm Hg systolic and/or 15 mm Hg rise in diastolic over the basal values.

## Conclusion

This study shows that teenage pregnancies are at risk of eclampsia-related maternal deaths and that attention to slight rises in BP from baseline might have prevented some deaths.
